# Response to ‘Methodological and statistical concerns in MINERVA microbiome–disease knowledge graph’ by Salvatore Chirumbolo

**DOI:** 10.1093/bib/bbaf671

**Published:** 2025-12-15

**Authors:** Saul Langarica, Young-Tak Kim, Adham Alkhadrawi, Jung Bin Kim, Synho Do

**Affiliations:** Department of Electrical Engineering, Pontificia Universidad Católica de Chile, Av. Vicuña Mackenna 4860, Macul, 7820436, Santiago, Región Metropolitana, Chile; Department of Radiology, Massachusetts General Hospital and Harvard Medical School, 125 Nashua Street, Boston, MA 02114, United States; Department of Radiology, Massachusetts General Hospital and Harvard Medical School, 125 Nashua Street, Boston, MA 02114, United States; Department of Radiology, Massachusetts General Hospital and Harvard Medical School, 125 Nashua Street, Boston, MA 02114, United States; Department of Molecular Biosciences and Bioengineering, University of Hawaii at Manoa, 2500 Campus Road, Honolulu, HI 96822, United States; Department of Neurology, Korea University Anam Hospital, Korea University College of Medicine, 73 Goryeodae-ro, Seongbuk-gu, Seoul 02841, Republic of Korea; Smart Healthcare Research Center, Korea University Anam Hospital, Korea University Medicine, 73 Goryeodae-ro, Seongbuk-gu, Seoul 02841, Republic of Korea; Artificial Intelligence R&D Center, Korea University Anam Hospital, Korea University Medicine, 73 Goryeodae-ro, Seongbuk-gu, Seoul 02841, Republic of Korea; Department of Radiology, Massachusetts General Hospital and Harvard Medical School, 125 Nashua Street, Boston, MA 02114, United States; KU-KIST Graduate School of Converging Science and Technology, Korea University, 145 Anam-ro, Seongbuk-gu, Seoul 02841, Republic of Korea; Kempner Institute, Harvard University, 150 Western Ave, Boston, MA 02134, United States

**Keywords:** microbiome, large language models, knowledge graph, ontology, gut microbes

We appreciate Dr Chirumbolo’s thoughtful evaluation and interest in our paper and welcome the opportunity to clarify MINERVA’s design choices and methodological rationale. Below, we address each point in detail.

## On the use of open-access literature

We fully acknowledge the limitation of relying exclusively on open-access sources such as PubMed and PubMed Central, as ‘explicitly stated’ in the Limitations and Future Work sections of our paper. This decision was driven by the need to ensure reproducibility, transparency, and unrestricted data accessibility.

In the Limitations section, we noted:

“Despite these strengths, users should remain aware of important limitations. The platform currently relies on open-access abstracts and full-text articles from PubMed and PubMed Central, which may introduce selection bias by excluding content from paywalled or non-indexed journals”.

In the *Future Work* section, we further emphasized our plan to address this constraint:

“To address these limitations and advance MINERVA, we will pursue several strategic directions. First, we aim to expand the knowledge base by incorporating additional repositories beyond PubMed, including clinical trial databases, longitudinal metagenomics datasets, and metabolomics resources. This will reduce bias and foster a more holistic understanding of microbiome compositions and their health implications”

Therefore, while we recognize that a certain degree of selection bias is inherent to the current implementation, this was a decision in favor of reproducibility, accessibility, and verifiable evidence, rather than an oversight. Currently, we are actively integrating additional data sources and repositories to minimize this bias and ensure that future releases of MINERVA offer a more comprehensive and balanced representation of the microbiome–disease landscape.

## On sentence-level relation extraction

In this case, we also ‘explicitly acknowledge’ this limitation in both the *Limitations* and *Future Work* sections of our paper, as we are fully aware that sentence-level relation extraction does not always capture broader discourse context or co-reference dependencies.

In the Limitations section, we stated:

“In addition, our pipeline focuses on extracting relationships at the sentence level. This may overlook more complex associations that span multiple sentences or require co-reference resolution and discourse-level analysis, an area we plan to address in future iterations”.

In the Future work section, we further noted:

“We also plan to enhance the NLP pipeline by implementing cross-sentence relationship extraction and coreference resolution to capture more complex associations”.

Nevertheless, we would like to note that this approach remains a widely adopted and peer-reviewed standard in biomedical natural language processing, adopted by established resources such as PubTator, SemMedDB, and several methods cited in our manuscript [1, 2]. These sentence-level models have proven to be reliable and scalable for large-scale literature mining, where interpretability and computational efficiency are critical.

However, to mitigate potential false positives arising from isolated sentence interpretation, MINERVA incorporates several safeguards. Specifically, only full-confidence predictions from our fine-tuned BioMistral-AUG-7b model were included in the knowledge graph, ensuring complete internal consistency across inference runs. In addition, when multiple sentences within the same publication described a microbe–disease pair with differing relation labels, a majority-voting mechanism was used to determine the final association (Methods, Fig. 6B). Furthermore, when multiple publications referred to the same association, we explicitly calculated a relationship strength score that aggregated and weighted the evidence across studies. Finally, all extracted relations are traceable to their exact supporting sentences, providing transparency, and enabling researchers to independently assess the evidence. This combination of strict confidence filtering, redundancy validation, and evidence traceability substantially reduces the likelihood of spurious relationships.

While we agree that incorporating cross-sentence and document-level reasoning would further enrich contextual understanding, we believe that the current implementation is methodologically robust, aligned with best practices in the field, and well suited for large-scale, explainable knowledge graph construction. Nonetheless, as noted in the Future Work section, we are actively developing extensions that integrate cross-sentence relation extraction and co-reference resolution in future versions of MINERVA.

## On journal impact factor as a weighting component

Again, we acknowledge the limitation of using the journal impact factor (IF) as the sole element in our weighting scheme and ‘explicitly addressed’ this in both the Limitations and Future Work sections of the paper.

In the Limitations section we stated:

*The weighting mechanism, which prioritizes information from high-impact journals, does not currently account for other study quality indicators such as sample size, replication, or methodological rigor.*

In the Future work section, we further noted:

*Additionally, we will develop a refined weighting strategy incorporating factors like study size, methodological rigor, and replication status, moving beyond journal impact factors alone.*

However, it is important to clarify that the IF influences only the relationship strength score, not the inclusion or the exclusion of relationships within the knowledge graph. All associations must first satisfy strict confidence thresholds from our relation extraction model and redundancy validation across multiple sources before any weighting is applied. Consequently, while some degree of bias related to journal IF may exist, its effect on the overall graph structure and content is limited.

Moreover, the inclusion of the IF was a deliberate and transparent design choice intended to modestly prioritize (logarithmic weighting) relationships consistently reported in journals with higher peer-review stringency, while maintaining a comprehensive representation of evidence from all accessible sources.

As acknowledged in the Future Work section, we are currently developing a more comprehensive and robust weighting framework that integrates additional quality metrics to better reflect the reliability of the underlying studies and further minimize potential bias.

## On link prediction model accuracy

We acknowledge that current performance of our prediction model (F1 ~ 71%) is limited and requires further improvement. We again, ‘explicitly acknowledge’ this limitation in the *Knowledge discovery* section, as follows:

“The current implementation uses Node2vec embeddings, achieving an accuracy and F1-score of ∼71% on a held-out test set. The remaining ∼30% of misclassifications have important implications: false positives may lead to spurious associations, while false negatives could obscure meaningful ones. As such, predictions from this module should be viewed as hypothesis-generating rather than definitive. We encourage users to validate these findings experimentally or clinically. Future versions of MINERVA will focus on improving performance through richer node features and more advanced graph learning architectures”.

As mentioned in the manuscript, the Link Prediction module is intended to assist hypothesis generation, not clinical decision-making.

## On risk scoring

We understand the concern raised in this point, however, we would like to clarify that MINERVA’s risk score was designed as a literature-based heuristic, rather than as a statistical estimator. Its purpose is to reflect the *direction* and *frequency* of associations reported across studies, independent of absolute abundance values or compositional transformations. To avoid any misunderstanding, ‘we explicitly defined’ this in the Risk Assessment Module section of the paper:

“In this context, ‘risk’ refers to a heuristic score that quantifies the cumulative literature-based evidence linking microbial imbalances to specific diseases. Microbial imbalances are defined as taxa whose relative abundances fall outside a configurable reference range (default: 5th–95th percentile) when compared to a healthy population. For each such imbalance, MINERVA queries its knowledge graph to determine whether the imbalance of this specific taxon is positively or negatively associated with any disease. A disease’s risk score is then calculated by summing +1 for each positively associated taxon and −1 for each negatively associated one. This simple yet interpretable scoring system reflects the direction and magnitude of literature-supported associations, offering insights into potential health concerns based on microbiome composition”.

As the description in the manuscript indicates, the risk score is intended to provide a qualitative, literature-grounded indicator of potential microbiome–disease relationships rather than a quantitative measure of clinical or statistical risk. It is therefore used to support hypothesis generation and exploratory analysis, not diagnostic or inferential conclusions.

## On multiple testing and false discovery rate

We agree that formal FDR control is crucial in hypothesis-driven statistical testing. However, MINERVA’s relation extraction is deterministic once evidence thresholds are met; no inferential p-values are computed. Therefore, traditional FDR corrections are not directly applicable.

In conclusion, we sincerely appreciate Dr Chirumbolo’s thoughtful engagement with our work and the opportunity to further clarify MINERVA’s methodology. We respectfully note, however, that most of the points raised do not directly apply or correspond to limitations that were already explicitly acknowledged and discussed in detail in our Limitations and Future Work sections. These aspects were transparently presented and carefully evaluated during the peer-review process prior to publication. Our goal has always been to communicate these constraints openly while outlining clear strategies for their resolution. We therefore view this exchange as a valuable opportunity to reaffirm our commitment to continuous improvement and to the ongoing development of MINERVA as a transparent, reliable, and evolving resource for the microbiome research community.

**Key Points**

- The methodological limitations highlighted in the critique were already explicitly acknowledged and discussed in the original manuscript’s Limitations and Future Work sections, where we outlined their scope, implications, and planned strategies for improvement.
- The design choices underlying MINERVA reflect established best practices in biomedical natural language processing and knowledge graph construction, and are supported by multiple safeguards, including full-confidence filtering, redundancy validation, and sentence-level evidence traceability, to ensure accuracy, transparency, and robustness.
- We remain committed to advancing and strengthening the MINERVA platform, and many of the improvements suggested, such as incorporation of additional data sources, enhanced contextual NLP modeling, and refined evidence weighting, are already part of ongoing development efforts aimed at further enriching the resource.

## Data Availability

All data, and code for reproducibility is available at https://github.com/MGH-LMIC/MINERVA. MINERVA’s platform is freely available at https://minervabio.org/. Additionally, all the entities and found relationships are available as a supplementary material in the main manuscript.
